# The Link between Trace Metal Elements and Glucose Metabolism: Evidence from Zinc, Copper, Iron, and Manganese-Mediated Metabolic Regulation

**DOI:** 10.3390/metabo13101048

**Published:** 2023-10-02

**Authors:** Zhendong Sun, Yuzhuo Shao, Kunhao Yan, Tianzhao Yao, Lulu Liu, Feifei Sun, Jiarui Wu, Yunpeng Huang

**Affiliations:** 1 Key Laboratory of Systems Health Science of Zhejiang Province, School of Life Science, Hangzhou Institute for Advanced Study, University of Chinese Academy of Sciences, Hangzhou 310024, China; 2 Key Laboratory of Systems Biology, Hangzhou Institute for Advanced Study, University of Chinese Academy of Sciences, Chinese Academy of Sciences, Hangzhou 310024, China

**Keywords:** trace metal elements, zinc, iron, copper, manganese, glucose metabolism, diabetes, insulin signaling

## Abstract

Trace metal elements are of vital importance for fundamental biological processes. They function in various metabolic pathways after the long evolution of living organisms. Glucose is considered to be one of the main sources of biological energy that supports biological activities, and its metabolism is tightly regulated by trace metal elements such as iron, zinc, copper, and manganese. However, there is still a lack of understanding of the regulation of glucose metabolism by trace metal elements. In particular, the underlying mechanism of action remains to be elucidated. In this review, we summarize the current concepts and progress linking trace metal elements and glucose metabolism, particularly for the trace metal elements zinc, copper, manganese, and iron.

## 1. Introduction

Trace metal elements, which are minerals present in living organisms in trace amounts, play an indispensable role in maintaining human health. In general, they perform crucial functions in various biological processes and exert significant influences on a wide range of biological events, including development, growth, physiology, aging, as well as the onset and progression of human diseases [1,2,3,4]. They function as the catalysts and co-factors in several enzymes, serving as structural stabilizers by means of incorporation and acting as electron acceptors in redox reactions [5]. For instance, zinc serves as a co-factor for over 300 enzymes and acts as a regulator for zinc finger-containing proteins that possess the Cys2His2, Cys4, and Cys6 motifs [6]. Copper serves as a cofactor in various copper-containing enzymes, including ceruloplasmin, cytochrome c oxidase, zinc-copper superoxide dismutase, and dopamine monooxygenase. In turn, it exerts an influence on iron metabolism, electron transport, oxidative phosphorylation (OXPHOS), antioxidant defense, and the synthesis of neurotransmitters [7]. Manganese serves as a vital co-factor for manganese superoxide dismutase (MnSOD), participating in antioxidant defense. Additionally, it acts as a co-factor for several enzymes, including arginase, glutamine synthetase (GS), and pyruvate carboxylase (PC) [8], contributing to the facilitation of development, digestion, and the immune response [8]. Imbalances in trace metal elements have been linked to several human diseases, including anemia, Wilson’s disease, atherosclerosis, gastrointestinal disorders, and diabetes [5,6,9]. The availability of trace metal elements is also critical for regulating cellular metabolisms such as lipid metabolism, glucose utilization and energy production, and amino acid synthesis [10].

Carbohydrates, predominantly glucose, are initially and most rapidly utilized during periods of energy shortage, thus making them the primary source of biological energy in living organisms. The normal range of glucose concentration in human peripheral blood is 3.9 to 5.5 mM and exceeds 5.5 mM in a fed state. Low plasma glucose levels can cause hypoglycemia, a condition that affects brain function and can lead to dizziness and coma. Conversely, high plasma glucose levels are associated with the development of diabetes and obesity [11]. Therefore, glucose metabolism is intricately regulated to maintain human health and homeostasis of glucose and energy production. Glycogenesis is the predominant regulatory process in the fed state, while glycogenolysis and gluconeogenesis are most important in starvation states. Moreover, glucose metabolism is tightly intertwined with a number of biological processes that govern its regulation, including the insulin signaling axis, plasma glucose uptake, glycolysis, the tricarboxylic acid (TCA) cycle, and OXPHOS [11,12].

Among the trace metal elements in the human body, iron is the most abundant and plays a crucial role in glucose metabolism. It is intricately involved in various metabolic processes through its incorporation into iron and iron-sulfur clusters-containing enzymes, such as aconitase and cytochromes [13]. Moreover, it actively participates in electron transfer processes and substrate oxidation-reduction reactions [14]. In addition to iron, other metal elements such as zinc, copper, and manganese also play a role in glucose metabolism by exerting diverse mechanisms that encompass the regulation of insulin synthesis and secretion, the modulation of glycolysis and TCA cycle activity, as well as the facilitation of glucose transportation [15,16,17,18,19,20]. In particular, the dysregulation of trace metal elements has been associated with high levels of plasma glucose and the development of diabetes (Table 1). However, the precise mechanisms underlying the relationship between trace metal elements and glucose metabolism regulation remain to be fully elucidated. Herein, we provide a summary of current research progress on the influences of trace metal elements (zinc, copper, manganese, and iron) on various aspects of glucose metabolism, including glycolysis, TCA cycle activity, electron transport chain (ETC) function, insulin production, and signaling pathways. These findings contribute to a better understanding of how trace metal elements impact glucose utilization and metabolism.

## 2. Zinc and Glucose Metabolism

The human body contains approximately 2 g of zinc, with only 0.1% present in the plasma and the remaining portion localized in cells [27]. The intracellular and intercellular distribution of zinc is tightly regulated by zinc transporters and zinc-binding proteins [28]. Zinc imbalances have been implicated in a number of human diseases, including obesity, insulin resistance, and type 2 diabetes (T2D) (Table 2 and Table S1) [29]. Intriguingly, mutations in ZnT8 have been implicated in glucose intolerance and T2D. Furthermore, ZnT8 has also been identified as an auto-antigen in type 1 diabetes (T1D) [30]. Additionally, significant associations have been observed between polymorphisms in the zinc-binding proteins metallothionein 1A and 2A and T2D [31]. Significantly, sufficient zinc intake has been demonstrated to mitigate the risk of developing diabetes [32].

Studies conducted in mice and HepG2 cells have demonstrated that zinc exerts multiple effects on the regulation of glucose metabolism. The severity of diabetes is associated with reduced plasma zinc levels, which are significantly diminished in individuals with diabetes [33]. In particular, a correlation has been observed between zinc deficiency and impaired plasma glucose levels in individuals with T2D [34]. Furthermore, zinc supplementation has demonstrated curative effects on diabetes [35], including the correction of glycaemia [36], improvements in pancreatic cell function, and the promotion of insulin synthesis and secretion [37,38]. In pancreatic β-cells, zinc has been proposed to enhance insulin secretion by binding to insulin and forming the hexametric complex within the secretory vesicles containing insulin [39]. ZnT8 is involved in transporting within secretory vesicles containing insulin [40]. Additionally, the involvement of ZnT5 and ZnT6 in zinc transportation towards secretory granules is also anticipated [41].

In addition to impacting insulin secretion, zinc also exerts a regulatory effect on the activity of the insulin signaling pathway by interacting with its components. For instance, in mice, zinc enhances phosphorylation of the β-subunit of the insulin receptor (InR) through binding and inhibiting protein tyrosine phosphatase (PTPase) 1B activity [42]. Since PTPase is a phosphatase that dephosphorylates the InR [42], inhibiting PTPase leads to the activation of Pi3K and PKB\AKT [43]. In addition, zinc exerts regulatory control over the degradation and expression of phosphatase and tensin homolog deleted on chromosome 10 (PTEN), thereby facilitating the activation of PKB\AKT [43]. As PKB\AKT phosphorylates and inhibits GSK3 activity, zinc activates the GLUT4-1 trafficking regulator (TRARG1), thereby promoting GLUT4 translocation to the membrane and facilitating glucose uptake [44]. The phosphorylation and subsequent inhibition of GSK3 can elicit the activation of GS [45]. The activation of the insulin signaling pathway also induces the down-regulation of phosphoenolpyruvate carboxykinase (PEPCK) and glucose-6-phosphatase (G6Pase), accompanied by an augmentation in glycogen synthesis [46]. The activation of insulin signaling can induce phosphorylation and subsequent inactivation of the forkhead box O (FOXO) transcription factor, thereby down-regulating the expression of gluconeogenic genes. Consequently, zinc possesses the capacity to attenuate gluconeogenesis activity in mice [47].

Through the regulation of glycolysis and the TCA cycle, zinc exerts a modulatory effect on energy production. Results obtained from rats indicate that zinc can stimulate glycolysis by activating glycolytic enzymes, such as phosphofructokinase 1 (PFK1) [48]. The stimulation of glycolysis by zinc in mouse models is suggested to be reliant on the formation of a complex between zinc and metallothionein (MT), which can potentially serve as a source of intracellular zinc donation [49]. Results from fungi indicate that zinc can modulate the activity of the TCA cycle. Notably, zinc deficiency maximizes the enzymatic activity of the TCA cycle, while adequate zinc levels also increase the activity of TCA cycle enzymes [50]. Although the underlying mechanism is not clear, zinc also possesses an impact on the activity of OXPHOS [51,52]. However, beyond physiologically tolerable concentrations, particularly at toxic concentrations, zinc exhibits inhibitory effects on both glycolysis and the TCA cycle. This phenomenon can be attributed to the inhibition of key enzymatic activities, including PFK, glyceraldehyde-3-phosphate dehydrogenase (GAPDH), α-ketoglutarate dehydrogenase complex (KGDHC), pyruvate dehydrogenase complex (PDHC), and mitochondrial aconitase (m-aconitase) [53]. These findings suggest that excessive zinc exposure has the potential to damage the entire energy production network.

**Table 2:** Trace metal elements (Zn, Cu, Mn, Fe) and diabetes.

Metal Elements	Association with Diabetes Mellitus	References
Zinc	Plasma zinc levels are decreased in diabetes. Increasing zinc levels reduces the risk of T2D ^1^. Low zinc levels aggravate diabetes. Zinc has curative effects on diabetes.	[33,35]
Copper	Plasma copper levels are increased in people with diabetes. High plasma copper levels increase the risk of T2D. Copper reduction can alleviate diabetic symptoms.	[54,55]
Manganese	Plasma manganese levels are reduced in T2D patients. Manganese supplementation can effectively protect against T2D.	[56,57]
Iron	Iron and ferritin levels are increased in diabetes. Iron overload increases the incidence of T2D. Iron depletion restores insulin sensitivity and reduces plasma glucose levels.	[58,59,60]

^1^ T2D: type 2 diabetes.

## 3. Copper and Glucose Metabolism

Copper is an indispensable trace metal element for living organisms and is ubiquitously present across almost all organisms. As a transition metal, copper exhibits dual oxidative states, and exists in both the oxidative form (Cu(II)) and reductive form (Cu(I)) [61]. Copper acts as a co-factor for metabolic enzymes involved in gluconeogenesis, lipids metabolism, and amino acid metabolism. In particular, copper plays a crucial role in the regulation of glucose utilization and is intricately involved in aerobic respiration, OXPHOS, and the ETC [62].

Copper plays a dual role in biological systems, as it is both indispensable and potentially detrimental. Copper deficiency tends to increase glycolytic fluxes and conversely inhibits the TCA cycle and the pentose phosphate pathway (PPP). Given the disruption of the ETC and ATP generation caused by copper deficiency, it is plausible that aerobic glycolysis is induced as a compensatory mechanism for energy production in mice and erythropoietic cells [63,64,65]. Furthermore, copper deficiency in *Trichoderma harzianum* not only results in a reduction in succinate dehydrogenase (SDH) activity but also hampers iron uptake [66].

In particular, copper has been shown to increase the production of ATP and NADH. Excessive copper can impede the activity of glycolysis by interfering with the activity of the glycolytic enzymes in Wilson’s disease rat model and in *Estuarine crab* [67,68]. The production of lactate from glucose and glucose-6-phosphate (G-6-P) is diminished upon copper treatment, concomitant with the copper-induced inhibition of hexokinase (HK) and pyruvate kinase (PK) [67]. Excessive copper also reduces pyruvate levels by inactivating PFK1 in eukaryotes and Archaea [69]. In addition, copper exhibits inhibitory effects on the enzymatic activity of glucose 6-phosphate dehydrogenase (G6PD) and 6-phosphogluconate dehydrogenase (6PGDH) [69,70]. Hence, it is plausible that copper exerts a negative regulatory effect on glycolytic activity.

In addition to inhibiting glycolysis, excess copper also leads to a decrease in citrate levels due to its ability to attenuate the activity of citrate synthase (CS) [70]. Excess copper levels also reduce the expression of isocitrate dehydrogenase (IDH) and alpha-ketoglutarate dehydrogenase (α-KGDH), thereby disrupting the TCA cycle and giving rise to metabolic disorders [71]. Interestingly, a recent study suggests that the disruption of the TCA cycle may also be attributed to copper binding with TCA cycle enzymes. Excess copper is able to induce cell death by directly interacting with lipoylated components of the TCA cycle, thereby promoting protein aggregation and the subsequent loss of iron-sulfur cluster proteins [72]. In addition to directly affecting glycolysis and the TCA cycle, the copper-induced disruption of iron uptake and heme synthesis may also contribute to metabolic perturbation in human cell culture and rat models [73,74,75].

It is worth noting that there exists an association between copper levels and diabetes (Table 1 and Table S1). Furthermore, copper-related genes have also been implicated in the pathogenesis of diabetes [76]. There is evidence indicating an increase in serum copper and ceruloplasmin levels in individuals with T1D [54,77]. Elevated plasma copper levels have been associated with a higher risk of developing T2D and hyperglycemia in humans [55]. In addition, the severity of T2D can be reduced by employing copper chelators to lower copper levels [78,79], which also corrects insulin resistance and glucose intolerance in diabetic animals [80].

It is worth noting that copper has the ability to activate insulin signaling, which operates independently of insulin and InR. Copper enhances downstream Pi3K and AKT activity, resulting in GSK3 phosphorylation and subsequent inactivation. Additionally, copper facilitates FOXO phosphorylation and translocation in HepG2 and HeLa cells [81,82,83]. The activation of Pi3K\AKT by copper is likely attributed to the copper-induced inactivation of PTPase, leading to downstream signaling activation [84,85]. Moreover, copper can decrease PTEN protein levels in the adipocytes of mice, which behave as the negative regulator of insulin signaling; thus, copper deficiency can lead to insulin resistance [86].

By modulating the expression of gluconeogenesis-related enzymes, particularly G6Pase, the activation of the insulin signaling pathway can effectively attenuate gluconeogenesis [81,87]. It is intriguing that the reduction of copper can alleviate the symptoms of T2D and inhibit insulin signaling, potentially due to the aberrant distribution of copper in T2D. Moreover, both physiological levels and excessive amounts of copper may disturb distinct physiological processes.

In addition to directly activating insulin signaling, copper also exhibits the ability to inhibit the activity of insulin-degrading enzymes (IDE), thereby impeding the degradation of extracellular insulin and consequently increasing insulin levels [88,89,90]. By modulating IDE function, copper is able to regulate plasma glucose levels, akin to the impact of IDE inhibitors in mice models [91].

The cellular uptake of glucose is also modulated by copper. In CHO cells, copper can increase the expression of glucose transporter type 1 (GLUT1) in a hypoxia-inducible factor-1 alpha (HIF-1α)-dependent manner, thereby augmenting glucose uptake [92]. Notably, copper is essential for the expression of a group of HIF-1α-regulated genes, including vascular endothelial growth factor (VEGF), GAPDH, GLUT1, and phosphoglycerate kinase 1 (PGK1). This is attributed to the ability of copper to modulate the binding affinity between HIF-1α and the canonical motifs in the promoter and enhancer regions [93].

Interestingly, copper depletion also stimulates glucose uptake and increases glycolysis in cancer cells. Given that copper depletion down-regulates the activity of cytochrome c oxidase (CCO) and ATP production associated with OXPHOS, the increase in glucose uptake can be attributed to the accelerated utilization of glucose by cancer cells [94].

The integration of copper into the ETC is indispensable for ETC assembly and exerts a significant influence on the enzymatic activity of the ETC components. In particular, copper ions are incorporated into the subunits of CCO to facilitate electron transfer and oxygen reduction to water. Within the catalytic center of the CCO complex, two copper ions are incorporated into CuA, while CuB incorporates one [17]. In addition, the assembly of CCO is intricately regulated by copper, and a deficiency in copper also leads to diminished levels of CCO proteins, consequently impeding their proper assembly [65]. Copper deficiency impairs ETC function, leading to reduced mitochondrial respiration, OXPHOS, and ATP production [95,96]. Conversely, increasing copper levels can increase the expression of ETC components and their assembly in K562 cells, resulting in an elevation in mitochondrial respiration and ATP production [97].

## 4. Manganese and Glucose Metabolism

Manganese plays a crucial role in various physiological processes, encompassing ATP synthesis, antioxidant defense, and the modulation of glycolysis [98]. Although the underlying mechanisms governing manganese homeostasis remain to be elucidated, both manganese deficiency and excess can disrupt glucose metabolism, potentially leading to hypoglycemia with chronic exposure. The oral intake of manganese in diabetic patients has been linked to decreased blood glucose levels [99]. The plasma manganese levels exhibit a reduction in T2D patients and demonstrate a significant correlation with T2D (Table 1). However, the role of manganese in the development of T2D remains uncertain [56,100]. The association between manganese and diabetes has been demonstrated to follow a U-shaped pattern, indicating that both deficiencies and excesses of manganese may be linked to the development of T2D [18,57]. Interestingly, MnSOD serves as a potential molecular bridge linking manganese and diabetes due to its capability to scavenge redox species that are abundant in T2D. In particular, the MnSOD Ala16Val SNP has been linked with the progression of T2D [101]. Manganese supplementation and increasing the activity of MnSOD have been demonstrated to confer significant protection against T2D in animal models [102,103].

It is noteworthy that manganese is able to regulate insulin production and secretion, thereby stimulating insulin signaling activity. In rats, a deficiency in manganese results in impaired insulin secretion and glucose intolerance [104]. The supplementation of manganese has been shown to enhance insulin secretion and ameliorate glucose intolerance in diabetic mice [102]. This effect is attributed to the stimulation of insulin synthesis and release by manganese [105].

In addition, manganese exhibits insulin-mimicking effects by modulating the activation of InR and the IGF receptor (IGF-R), thereby stimulating insulin signaling. Manganese achieves InR activation through a reduction in Km for Mg-ATP binding, subsequently leading to Pi3K-dependent AKT activation in a manner in adipocytes and cell culture models [106,107,108], and enhances glucose uptake [107]. In contrast, a deficiency in manganese reduces insulin signaling activity and glucose uptake in rat adipocytes [109]. Interestingly, recent evidence also suggests that manganese serves as a physiological activator of the target of rapamycin complex 1 (TORC1) in yeast and mammalian cell culture models [110], thereby potentially influencing the TORC1-mediated regulation of glucose metabolism [111].

Manganese exhibits the ability to impede the activity of HK and PK in human neuroblastoma (SK-N-SH) and astrocytoma (U87) cells [112], thereby resulting in the repression of glycolysis. Additionally, manganese also suppresses the lactate-induced activation of PC and phosphoenolpyruvate carboxykinase (PCK1) in rat liver [113]. The manganese-containing compound known as manganese porphyrin (MnP) exhibits a propensity to attenuate glycolytic activity and lactate levels [114], thereby providing further evidence of the inhibitory effect of manganese on glycolysis.

Manganese also exerts an inhibitory effect on the TCA cycle, leading to a reduction in OXPHOS [115]. In particular, manganese competes with other metal elements for protein binding capacity, such as iron, thereby potentially interfering with iron’s role. This competition can result in a reduction in mitochondrial aconitase activity and hinder the conversion of citrate to isocitrate, ultimately disrupting the TCA cycle in the rat brain [116]. Furthermore, manganese possesses the capability to deplete iron from bacteria, thereby disrupting the synthesis of iron-sulfur clusters. Since the iron-sulfur clusters play a crucial role in the functional ETC, manganese can consequently attenuate OXHPOS and impede energy production in bacteria [117]. Evidence from mammalian cells also indicates that excessive manganese hampers the activity of the TCA cycle and impairs glucose utilization [115].

MnSOD exhibits a close association with glucose metabolism, and the underlying connection between them is likely facilitated by reactive oxygen species (ROS). In fact, ROS is not only a by-product of aerobic metabolism but also a pivotal signaling molecule [118]. For instance, ROS is able to induce the expression of MnSOD by activating NF-kappa B (NF-κB) during muscle differentiation [119]. In cancer cells, the up-regulation of MnSOD facilitates the sustained generation of hydrogen peroxide (H_2_O_2_), thereby enabling a metabolic switch towards glycolysis [58]. Therefore, the accumulation of ROS likely functions as a signaling molecule to regulate MnSOD expression, resulting in H_2_O_2_ accumulation and redirection of the TCA cycle towards glycolysis.

## 5. Iron and Glucose Metabolism

In comparison to zinc, copper, and manganese, the impact of iron on glucose metabolism and energy production has been extensively documented [120,121,122,123]. Iron plays a crucial role in modulating the activity and expression of TCA cycle-associated enzymes, including CS, mitochondrial aconitase, isocitrate dehydrogenase (ICDH) and SDH. Depletion of iron can impair the functionality of the TCA cycle in K562 cells [19].

Specifically, iron depletion diminishes the NADH level, which is generated from the TCA cycle and serves as an electron donor for OXPHOS. Consequently, iron can attenuate the activity of OXPHOS and ETC-mediated mitochondrial respiration [19]. The synthesis of cytochrome c (Cyt c) is also regulated by iron; therefore, iron deficiency leads to a reduction in Cyt c levels and the inhibition of ETC activity [124]. Reversely, iron supplementation enhances the activity of the TCA cycle, elevates NADH levels, augments OXPHOS, and boosts mitochondrial respiration [19]. In addition, iron deficiency also impacts oxygen transportation in rats since the iron-sulfur clusters are more vulnerable to fluctuations in iron levels, which are incorporated into hemoglobin and serve as oxygen carriers [125].

Compensating for the inhibition of the TCA cycle and OXPHOS, iron deficiency up-regulates glycolytic activity, resulting in an elevation of lactate levels and the promotion of lactate dehydrogenase (LDH) activity [19]. Iron deficiency has been linked to metabolic shifts that facilitate glycolysis in both human and animal models [126,127]. In particular, iron deficiency is able to induce the up-regulation of LDH expression and a series of glycolytic enzymes, including pyruvate dehydrogenase kinase 1 (PDK1) [127], the LDH isoenzyme [128], and GAPDH [129].

The up-regulation of iron levels in mice models can suppress hepatic gluconeogenesis by reducing the expression of key gluconeogenic enzymes, including PCK1 and G6Pase [130]. In contrast, iron deficiency up-regulates the expression of G6Pase and PCK1 in rats [131], indicating a negative association between iron status and gluconeogenesis and glucose turnover [132]. However, emerging evidence suggests that iron deficiency also exerts inhibitory effects on the activity of alpha-glycerophosphate cytochrome c reductase and PCK1 in isolated rat hepatocytes [59], thereby implying a multifaceted impact of iron.

Notably, accumulating evidence indicates that iron status is perturbed in individuals with diabetes, which is believed to be associated with insulin sensitivity [133]. Dietary iron intake is associated with an increased risk of diabetes [134]. Iron overload and related disorders have also been linked to an increased risk of diabetes. Specifically, hereditary hemochromatosis (HH) caused by HFE mutation and thalassemia resulting from beta-globin subunit deficiency in hemoglobin both contribute to elevated risks of diabetes and hyperglycemia [121]. Furthermore, the SLC7A11 rs3731685 G/A variation has been suggested to be correlated with T1D [135], while rs3731865 G/C may exhibit positive associations with a predisposition to T2DM; however, the rs3731864 G/A variation shows a negative association [136].

The levels of iron and ferritin exhibit abnormal elevation in individuals with diabetes [137]. Iron overload represents a risk factor that is associated with the incidence of T2D (Table 1) [121]. Elevated levels of body iron and ferritin, as well as dietary intake of heme, exhibit a significant association with the risk of T2D [122]. Increased plasma ferritin and body iron levels are associated with insulin resistance [137]. In particular, iron overload has been shown to adversely impact insulin sensitivity [138], whereas iron depletion has been found to restore insulin sensitivity and decrease the levels of plasma glucose [139], indicating a potential association between iron levels and insulin signaling activity.

In particular, iron possesses the capacity to disrupt insulin signaling activity. The excessive accumulation of iron in AML-12 hepatocytes results in the inhibition of insulin signaling, which can be ascribed to iron-induced oxidative stress and the down-regulation of InR expression and activity [140]. Iron depletion can ameliorate insulin resistance by augmenting the activity of insulin signaling through up-regulation of InR expression and activity [141,142]. In hepatoma cells and rat livers, the up-regulation of InR expression may be attributed to the stabilization of HIF-1α, as the hydroxylation and subsequent degradation of HIF-1α are tightly regulated by prolyl-4-hydroxylases that rely on iron and oxygen [142]. Besides InR, iron depletion also increases AKT expression, up-regulates GLUT1 in rat livers, and up-regulates both InR and GLUT4 in rat skeletal muscles. Thus, iron depletion promotes glucose uptake and glucose metabolism [142,143].

Moreover, iron possesses the capacity to disrupt insulin secretion in pancreatic cells. Excessive iron leads to a decrease in insulin secretion in MIN6-β cells, which is attributed to iron-induced oxidative stress and the down-regulation of synaptosomal associated protein 25 (SNAP25). Additionally, iron also disturbs the transcription of synaptotagmin 7 (SYT7), which is regulated by OGG1 [144]. Therefore, iron can disrupt the secretory pathway and attenuate insulin secretion [145]. Excessive iron has the capacity to impair islet β cells by activating the miR-130a-3p/ALK2 axis [146] and inducing ferroptosis through activation of the ASK1/P-P38/CHOP signaling pathway [147]. Therefore, the reduced insulin secretion can be attributed to both the inhibition of iron-induced cell death and the suppression of secretion.

Furthermore, the ablation of divalent metal transporter 1 (DMT1) in islet β cells also diminishes insulin production and secretion [148], implying that iron plays a crucial role in sustaining insulin synthesis. Additionally, iron regulatory protein 2 (IRP2) is also crucial for insulin production. The deletion of IRP2 in mice leads to impaired proinsulin processing due to the misreading of lysine residues. This is attributed to the inhibition of the iron-sulfur cluster-containing enzyme-CDK5 Regulatory Subunit Associated Protein 1 Like 1 (CDKAL1), which contributes to the translation of lysine residues on proinsulin by catalyzing tRNALys (UUU) methylthiolation [149].

By modulating insulin signaling, iron can also regulate glucose uptake by influencing the expression and translocation of glucose transporters. Iron depletion in pancreatic β cells leads to the up-regulation of GLUT2 and enhanced glucose uptake, whereas iron overload reduces HIF-1α levels, thereby inhibiting GLUT2 expression and impairing glucose uptake [150]. Iron depletion in skeletal muscle and myoblasts also increases glucose uptake, attributed to the up-regulation of GLUT4 and GLUT1 [15,143]. In addition, iron depletion in rat livers results in an up-regulation of GLUT1 expression, which is dependent on HIF-1α [142] and has been demonstrated in adipocytes as well. Interestingly, GLUT4 is down-regulated in iron-depleted adipocytes [151]. Therefore, it is plausible to propose that HIF-1α plays a crucial role in modulating the expression of glucose transporters influenced by iron, considering the negative regulatory effect of iron on HIF-1α stability [152].

In addition, considering the pivotal role of insulin signaling in regulating the translocation of glucose transporters, particularly GLUT4 translocation from intracellular vesicles to the cell membrane, it is plausible that iron-mediated insulin resistance and inactivation of insulin signaling may potentially modulate glucose uptake by exerting an influence on the translocation process [153,154].

## 6. Conclusions

**Figure 1:**
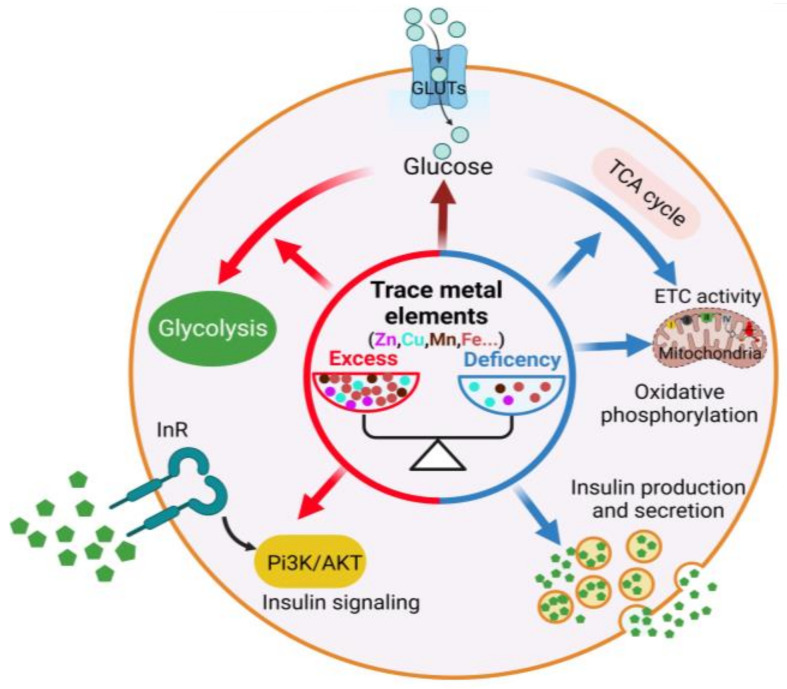
Trace metal elements and glucose metabolism.

The present review provides an overview of the influence of trace metal elements on glucose metabolism, highlighting their crucial role in the physiological regulation of this metabolic process. The dysregulation of trace metal elements is intricately linked to disruptions in glucose homeostasis, thereby contributing to the pathogenesis of related human disorders such as diabetes and hyperglycemia. They possess the capacity to modulate multiple regulatory steps, exert influence over insulin synthesis and secretion, regulate insulin signaling activity, modulate the activity of glycolysis and TCA cycle, as well as govern OXPHOS and glucose uptake (Figure 1). In particular, zinc is able to increase insulin synthesis and secretion, augment glucose uptake and glycolytic activity, diminish gluconeogenesis, as well as reduce the activity of TCA cycle and OXPHOS. Copper confers benefits on the TCA cycle and ETC activity and activates insulin signaling and glucose uptake while reducing gluconeogenesis and glycolysis. Manganese can stimulate insulin production and secretion, promote the activity of insulin signaling and glucose uptake, and suppress TCA cycle and OXPHOS while concurrently redirecting the TCA cycle towards glycolysis by up-regulating MnSOD levels. Iron facilitates ETC assembly and activity, increases OXPHOS, and reduces gluconeogenesis and glucose uptake while impeding insulin secretion and the activity of insulin signaling. Thereby, modulating the levels of trace metal elements holds the potential for curating glucose metabolism-related disorders. Indeed, increasing evidence suggests that manipulating trace metal elements indeed has the capacity to intervene in diabetes (Supplementary Table S1).

In addition to directly impacting the glucose metabolic pathway, the interplay and competition among the trace metal elements also contribute to the metabolic interventions of glucose. Although the modulation of cellular and animal models’ trace metal element homeostasis has been proposed as a promising strategy for intervening in glucose metabolism and associated disorders (Supplementary Table S1), the current lack of compelling clinical trials remains evident. Furthermore, the dysregulation of metal homeostasis in glucose metabolic disorders may serve as both a causative factor and a consequential outcome; however, the precise interrelationship remains to be elucidated. It is worth noting that both deficiency and excess of trace metal elements can disrupt the metabolic process of glucose, resulting in detrimental effects.

Henceforth, the future consideration of confining metal concentrations within a physiologically tolerable range is warranted [155,156,157]. Moreover, it is imperative to devise methodologies capable of precisely modulating the concentration of trace metal elements within a confined region and specific cell types and organs, thereby circumventing any systemic repercussions subsequent to administration. However, tissue- and cell-type-specific metal chelators and deliveries are still lacking and deserve to be developed further, which is significant for the metal-based intervention of diabetes and glucose-related metabolic disorders.

## Figures and Tables

**Table 1:** The concentrations of Zn, Cu, Mn, and Fe in subjects with and without diabetes.

Sample	Trace Metal Elements	Concentration		References
		Healthy People	Diabetic Patients	
Plasma	Zinc	12.2–16.9 μmol/L	10.2–16.3 μmol/L	[21,22,23]
	Copper	14.6–19.8 μmol/L	16.4–21.5 μmol/L	[22]
	Manganese	0.77–1.18 μmol/L	0.62–0.93 μmol/L	[22,23,24,25]
	Iron	Male: 11–31 μmol/L, Female: 9–30 μmol/L	Male: 15–33 μmol/L Female: 12–32 μmol/L	[24,26]
Urine	Zinc	300–600 μg/24 h ^1^	100–300 μg/24 h	[24]
	Copper	15–60 μg/24 h	30–100 μg/24 h	[24]
	Manganese	1–8 μg/24 h	2–15 μg/24 h	[24,25]
	Iron	0.2–1.1 μmol/24 h	0.5–3.5 μmol/24 h	[26]

^1^ The 24-h urine specimens were collected.

## Supplementary Materials

The following supporting information can be downloaded at: https://www.mdpi.com/article/10.3390/metabo13101048/s1, Table S1: Implications of trace metal elements in diabetic therapy [158,159,160,161,162,163,164,165,166,167,168,169,170,171].

## Abbreviations

AKT\PKB	protein kinase B
ATP	adenosine triphosphate
ASK	Apoptosis Signal Regulating Kinase
Cdkal1	CDK5 Regulatory Subunit Associated Protein 1-like 1
COX	cytochrome c oxidase
CHOP	Human Endoplasmic reticulum stress-related proteins
CS	citrate synthase
Cyt c	cytochrome c
ETC	electron transport chain
G6Pase	glucose-6-phosphatase
GAPDH	glyceraldehyde-3-phosphate dehydrogenase
GlUT	glucose transporter
GS	glutamine synthetase
GSK	glycogen synthase kinase
H2O2	hydrogen peroxide
HIF-1α	hypoxia inducible factor-1
IDE	insulin-degrading enzymes
IGF-R	IGF receptor
InR	insulin receptor
IRP2	iron regulatory protein 2
KGDHC	α-ketoglutarate dehydrogenase complex
LDH	lactate dehydrogenase
MnP	Mn porphyrin
MnSOD	manganese superoxide dismutase
MT	metallothionein
NADH	nicotinamide adenine dinucleotide
NF-κB	NF-kappa B
OGG1	recombinant oxoguanine glycosylase 1
OXPHOS	oxidative phosphorylation
PCK1	phosphoenolpyruvate carboxykinase
PDHC	pyruvate dehydrogenase complex
PDK1	pyruvate dehydrogenase kinase 1
PFK1	phosphofructokinase 1
PGK1	phosphoglycerate kinase 1
Pi3K	phosphatidylinositol3-kinase
PPP	pentose phosphate pathway
PTEN	phosphatase and tensin
PTPase	protein tyrosine phosphatase
ROS	reactive oxygen species
SDH	succinate dehydrogenase
SNAP25	synaptosomal associated protein 25
SYT7	synaptotagmin 7
T2D	type 2 diabetes
TCA cycle	tricarboxylic acids cycle
TORC1	target of rapamycin complex 1
TRARG1	GLUT4-1 trafficking regulator 1
VEGF	vascular endothelial growth factor
ZnT	zinc transporter

## References

[1] Mertz W. (1981). The Essential Trace Elements. Science.

[2] Hänsch R., Mendel R.R. (2009). Physiological functions of mineral micronutrients (Cu, Zn, Mn, Fe, Ni, Mo, B, Cl). Curr. Opin. Plant Biol..

[3] He Z., Geng S., Pan Y., Cai C., Wang J., Wang L., Liu S., Zheng P., Xu X., Hu B. (2015). Improvement of the trace metal composition of medium for nitrite-dependent anaerobic methane oxidation bacteria: Iron (II) and copper (II) make a difference. Water Res..

[4] Wilson D. (2021). The role of zinc in the pathogenicity of human fungal pathogens. Adv. Appl. Microbiol..

[5] Islam M.R., Akash S., Jony M.H., Alam M.N., Nowrin F.T., Rahman M.M., Rauf A., Thiruvengadam M. (2023). Exploring the potential function of trace elements in human health: A therapeutic perspective. Mol. Cell. Biochem..

[6] Chasapis C.T., Loutsidou A.C., Spiliopoulou C.A., Stefanidou M.E. (2012). Zinc and human health: An update. Arch. Toxicol..

[7] Mehri A. (2020). Trace Elements in Human Nutrition (II)—An Update. Int. J. Prev. Med..

[8] Chen P., Bornhorst J., Aschner M. (2018). Manganese metabolism in humans. Front. Biosci..

[9] Shribman S., Poujois A., Bandmann O., Czlonkowska A., Warner T.T. (2021). Wilson’s disease: Update on pathogenesis, biomarkers and treatments. J. Neurol. Neurosurg. Psychiatry.

[10] Prabhu A., Gadgil M. (2021). Trace metals in cellular metabolism and their impact on recombinant protein production. Process Biochem..

[11] Chandel N.S. (2021). Carbohydrate Metabolism. Cold Spring Harb. Perspect. Biol..

[12] Norton L., Shannon C., Gastaldelli A., DeFronzo R.A. (2022). Insulin: The master regulator of glucose metabolism. Metabolism.

[13] Susnea I., Weiskirchen R. (2016). Trace metal imaging in diagnostic of hepatic metal disease. Mass Spectrom. Rev..

[14] Beard J.L. (2001). Iron biology in immune function, muscle metabolism and neuronal functioning. J. Nutr..

[15] Potashnik R., Kozlovsky N., Ben-Ezra S., Rudich A., Bashan N. (1995). Regulation of glucose transport and GLUT-1 expression by iron chelators in muscle cells in culture. Am. J. Physiol..

[16] Fukunaka A., Fujitani Y. (2018). Role of Zinc Homeostasis in the Pathogenesis of Diabetes and Obesity. Int. J. Mol. Sci..

[17] Ruiz L.M., Libedinsky A., Elorza A.A. (2021). Role of Copper on Mitochondrial Function and Metabolism. Front. Mol. Biosci..

[18] Li L., Yang X. (2018). The Essential Element Manganese, Oxidative Stress, and Metabolic Diseases: Links and Interactions. Oxid. Med. Cell. Longev..

[19] Oexle H., Gnaiger E., Weiss G. (1999). Iron-dependent changes in cellular energy metabolism: Influence on citric acid cycle and oxidative phosphorylation. Biochim. Biophys. Acta.

[20] Wiernsperger N., Rapin J. (2010). Trace elements in glucometabolic disorders: An update. Diabetol. Metab. Syndr..

[21] Al-Maroof R.A., Al-Sharbatti S.S. (2006). Serum zinc levels in diabetic patients and effect of zinc supplementation on glycemic control of type 2 diabetics. Saudi Med. J..

[22] Viktorínová A., Toserová E., Krizko M., Duracková Z. (2009). Altered metabolism of copper, zinc, and magnesium is associated with increased levels of glycated hemoglobin in patients with diabetes mellitus. Metab. Clin. Exp..

[23] Masood N., Baloch G.H., Ghori R.A., Memon I.A., Memon M.A., Memon M.S. (2009). Serum zinc and magnesium in type-2 diabetic patients. J. Coll. Physicians Surg.-Pak. JCPSP.

[24] Kazi T.G., Afridi H.I., Kazi N., Jamali M.K., Arain M.B., Jalbani N., Kandhro G.A. (2008). Copper, chromium, manganese, iron, nickel, and zinc levels in biological samples of diabetes mellitus patients. Biol. Trace Elem. Res..

[25] Uğurlu V., Binay Ç., Şimşek E., Bal C. (2016). Cellular Trace Element Changes in Type 1 Diabetes Patients. J. Clin. Res. Pediatr. Endocrinol..

[26] Zhou Q., Guo W., Jia Y., Xu J. (2019). Comparison of Chromium and Iron Distribution in Serum and Urine among Healthy People and Prediabetes and Diabetes Patients. BioMed Res. Int..

[27] Bafaro E., Liu Y., Xu Y., Dempski R.E. (2017). The emerging role of zinc transporters in cellular homeostasis and cancer. Signal Transduct. Target. Ther..

[28] Myers S.A., Nield A., Myers M. (2012). Zinc transporters, mechanisms of action and therapeutic utility: Implications for type 2 diabetes mellitus. J. Nutr. Metab..

[29] Roohani N., Hurrell R., Kelishadi R., Schulin R. (2013). Zinc and its importance for human health: An integrative review. J. Res. Med. Sci..

[30] Rungby J. (2010). Zinc, zinc transporters and diabetes. Diabetologia.

[31] Jansen J., Karges W., Rink L. (2009). Zinc and diabetes—Clinical links and molecular mechanisms. J. Nutr. Biochem..

[32] Fernández-Cao J.C., Warthon-Medina M., Moran V.H., Arija V., Doepking C., Serra-Majem L., Lowe N.M. (2019). Zinc Intake and Status and Risk of Type 2 Diabetes Mellitus: A Systematic Review and Meta-Analysis. Nutrients.

[33] Qi Y., Zhang Z., Liu S., Aluo Z., Zhang L., Yu L., Li Y., Song Z., Zhou L. (2020). Zinc Supplementation Alleviates Lipid and Glucose Metabolic Disorders Induced by a High-Fat Diet. J. Agric. Food Chem..

[34] Bandeira V.D.S., Pires L.V., Hashimoto L.L., Alencar L.L., Almondes K.G.S., Lottenberg S.A., Cozzolino S.M.F. (2017). Association of reduced zinc status with poor glycemic control in individuals with type 2 diabetes mellitus. J. Trace Elem. Med. Biol..

[35] Jayawardena R., Ranasinghe P., Galappatthy P., Malkanthi R., Constantine G., Katulanda P. (2012). Effects of zinc supplementation on diabetes mellitus: A systematic review and meta-analysis. Diabetol. Metab. Syndr..

[36] Wang X., Wu W., Zheng W., Fang X., Chen L., Rink L., Min J., Wang F. (2019). Zinc supplementation improves glycemic control for diabetes prevention and management: A systematic review and meta-analysis of randomized controlled trials. Am. J. Clin. Nutr..

[37] Nygaard S.B., Larsen A., Knuhtsen A., Rungby J., Smidt K. (2014). Effects of zinc supplementation and zinc chelation on in vitro β-cell function in INS-1E cells. BMC Res. Notes.

[38] Cooper-Capetini V., de Vasconcelos D.A.A., Martins A.R., Hirabara S.M., Donato J., Carpinelli A.R., Abdulkader F. (2017). Zinc Supplementation Improves Glucose Homeostasis in High Fat-Fed Mice by Enhancing Pancreatic β-Cell Function. Nutrients.

[39] Li Y.V. (2014). Zinc and insulin in pancreatic beta-cells. Endocrine.

[40] Chimienti F., Devergnas S., Favier A., Seve M. (2004). Identification and cloning of a β-cell-specific zinc transporter, ZnT-8, localized into insulin secretory granules. Diabetes.

[41] Cruz K.J.C., de Oliveira A.R.S., Morais J.B.S., Severo J.S., Mendes P.M.V., de Sousa Melo S.R., de Sousa G.S., Marreiro D.D.N. (2018). Zinc and Insulin Resistance: Biochemical and Molecular Aspects. Biol. Trace Elem. Res..

[42] Bellomo E., Massarotti A., Hogstrand C., Maret W. (2014). Zinc ions modulate protein tyrosine phosphatase 1B activity. Metallomics.

[43] Barthel A., Ostrakhovitch E.A., Walter P.L., Kampkötter A., Klotz L.O. (2007). Stimulation of phosphoinositide 3-kinase/Akt signaling by copper and zinc ions: Mechanisms and consequences. Arch. Biochem. Biophys..

[44] Duan X., Norris D.M., Humphrey S.J., Yang P., Cooke K.C., Bultitude W.P., Parker B.L., Conway O.J., Burchfield J.G., Krycer J.R. (2022). Trafficking regulator of GLUT4-1 (TRARG1) is a GSK3 substrate. Biochem. J..

[45] Wang L., Li J., Di L.J. (2022). Glycogen synthesis and beyond, a comprehensive review of GSK3 as a key regulator of metabolic pathways and a therapeutic target for treating metabolic diseases. Med. Res. Rev..

[46] Hall R.K., Wang X.L., George L., Koch S.R., Granner D.K. (2007). Insulin Represses Phosphoenolpyruvate Carboxykinase Gene Transcription by Causing the Rapid Disruption of an Active Transcription Complex: A Potential Epigenetic Effect. Mol. Endocrinol..

[47] Puigserver P., Rhee J., Donovan J., Walkey C.J., Yoon J.C., Oriente F., Kitamura Y., Altomonte J., Dong H., Accili D. (2003). Insulin-regulated hepatic gluconeogenesis through FOXO1-PGC-1α interaction. Nature.

[48] Tamaki N., Ikeda T., Funatsuka A. (1983). Zinc as activating cation for muscle glycolysis. J. Nutr. Sci. Vitaminol..

[49] Rofe A.M., Philcox J.C., Coyle P. (2000). Activation of glycolysis by zinc is diminished in hepatocytes from metallothionein-null mice. Biol. Trace Elem. Res..

[50] Gupta S.K., Maggon K.K., Venkitasubramanian T.A. (1977). Effect of Zinc on tricarboxylic acid cycle intermediates and enzymes in relation to aflatoxin biosynthesis. J. Gen. Microbiol..

[51] Yang X., Wang H., Huang C., He X., Xu W., Luo Y., Huang K. (2017). Zinc enhances the cellular energy supply to improve cell motility and restore impaired energetic metabolism in a toxic environment induced by OTA. Sci. Rep..

[52] Zhang G., Sheng M., Wang J., Teng T., Sun Y., Yang Q., Xu Z. (2018). Zinc improves mitochondrial respiratory function and prevents mitochondrial ROS generation at reperfusion by phosphorylating STAT3 at Ser(727). J. Mol. Cell. Cardiol..

[53] Liu H.Y., Gale J.R., Reynolds I.J., Weiss J.H., Aizenman E. (2021). The Multifaceted Roles of Zinc in Neuronal Mitochondrial Dysfunction. Biomedicines.

[54] Walter R.M., Uriu-Hare J.Y., Olin K.L., Oster M.H., Anawalt B.D., Critchfield J.W., Keen C.L. (1991). Copper, zinc, manganese, and magnesium status and complications of diabetes mellitus. Diabetes Care.

[55] Yin J., Wang X., Li S., Zhu Y., Chen S., Li P., Luo C., Huang Y., Li X., Hu X. (2019). Interactions between plasma copper concentrations and SOD1 gene polymorphism for impaired glucose regulation and type 2 diabetes. Redox Biol..

[56] Koh E.S., Kim S.J., Yoon H.E., Chung J.H., Chung S., Park C.W., Chang Y.S., Shin S.J. (2014). Association of blood manganese level with diabetes and renal dysfunction: A cross-sectional study of the Korean general population. BMC Endocr. Disord..

[57] Shan Z., Chen S., Sun T., Luo C., Guo Y., Yu X., Yang W., Hu F.B., Liu L. (2016). U-Shaped Association between Plasma Manganese Levels and Type 2 Diabetes. Environ. Health Perspect..

[58] Hart P.C., Mao M., de Abreu A.L., Ansenberger-Fricano K., Ekoue D.N., Ganini D., Kajdacsy-Balla A., Diamond A.M., Minshall R.D., Consolaro M.E. (2015). MnSOD upregulation sustains the Warburg effect via mitochondrial ROS and AMPK-dependent signalling in cancer. Nat. Commun..

[59] Klempa K.L., Willis W.T., Chengson R., Dallman P.R., Brooks G.A. (1989). Iron deficiency decreases gluconeogenesis in isolated rat hepatocytes. J. Appl. Physiol..

[60] Fernández-Real J.M., López-Bermejo A., Ricart W. (2002). Cross-talk between iron metabolism and diabetes. Diabetes.

[61] Vetchý M. (2018). Biological role of copper as an essential trace element in the human organism. Ceska Slov. Farm..

[62] Xue Q., Kang R., Klionsky D.J., Tang D., Liu J., Chen X. (2023). Copper metabolism in cell death and autophagy. Autophagy.

[63] Nargund S., Qiu J., Goudar C.T. (2015). Elucidating the role of copper in CHO cell energy metabolism using (13)C metabolic flux analysis. Biotechnol. Prog..

[64] Cui L., Gouw A.M., LaGory E.L., Guo S., Attarwala N., Tang Y., Qi J., Chen Y.S., Gao Z., Casey K.M. (2021). Mitochondrial copper depletion suppresses triple-negative breast cancer in mice. Nat. Biotechnol..

[65] Bustos R.I., Jensen E.L., Ruiz L.M., Rivera S., Ruiz S., Simon F., Riedel C., Ferrick D., Elorza A.A. (2013). Copper deficiency alters cell bioenergetics and induces mitochondrial fusion through up-regulation of MFN2 and OPA1 in erythropoietic cells. Biochem. Biophys. Res. Commun..

[66] Tavsan Z., Ayar Kayali H. (2013). The effect of iron and copper as an essential nutrient on mitochondrial electron transport system and lipid peroxidation in *Trichoderma harzianum*. Appl. Biochem. Biotechnol..

[67] Lai J.C., Blass J.P. (1984). Neurotoxic effects of copper: Inhibition of glycolysis and glycolytic enzymes. Neurochem. Res..

[68] Lauer M.M., de Oliveira C.B., Yano N.L., Bianchini A. (2012). Copper effects on key metabolic enzymes and mitochondrial membrane potential in gills of the estuarine crab *Neohelice granulata* at different salinities. Comp. Biochem. Physiol. Part C Toxicol. Pharmacol..

[69] Gebhard S., Ronimus R.S., Morgan H.W. (2001). Inhibition of phosphofructokinases by copper(II). FEMS Microbiol. Lett..

[70] Tavsan Z., Ayar Kayali H. (2015). The Variations of Glycolysis and TCA Cycle Intermediate Levels Grown in Iron and Copper Mediums of *Trichoderma harzianum*. Appl. Biochem. Biotechnol..

[71] Li X., Qin Y., Kong L., Yan X., Zhang W., Martyniuk C.J., Wang X., Yan B. (2022). Metabolomic and bioenergetic responses of human hepatocellular carcinoma cells following exposure to commercial copper hydroxide nanopesticide. Environ. Sci. Nano.

[72] Tsvetkov P., Coy S., Petrova B., Dreishpoon M., Verma A., Abdusamad M., Rossen J., Joesch-Cohen L., Humeidi R., Spangler R.D. (2022). Copper induces cell death by targeting lipoylated TCA cycle proteins. Science.

[73] Wazir S.M., Ghobrial I. (2017). Copper deficiency, a new triad: Anemia, leucopenia, and myeloneuropathy. J. Community Hosp. Intern. Med. Perspect..

[74] D’Angelo G. (2016). Copper deficiency mimicking myelodysplastic syndrome. Blood Res..

[75] Williams D.M., Kennedy F.S., Green B.G. (1985). The effect of iron substrate on mitochondrial haem synthesis in copper deficiency. Br. J. Nutr..

[76] Ming J., Sana S., Deng X. (2022). Identification of copper-related biomarkers and potential molecule mechanism in diabetic nephropathy. Front. Endocrinol..

[77] Cunningham J., Leffell M., Mearkle P., Harmatz P. (1995). Elevated plasma ceruloplasmin in insulin-dependent diabetes mellitus: Evidence for increased oxidative stress as a variable complication. Metabolism.

[78] Cooper G.J., Chan Y.K., Dissanayake A.M., Leahy F.E., Keogh G.F., Frampton C.M., Gamble G.D., Brunton D.H., Baker J.R., Poppitt S.D. (2005). Demonstration of a hyperglycemia-driven pathogenic abnormality of copper homeostasis in diabetes and its reversibility by selective chelation: Quantitative comparisons between the biology of copper and eight other nutritionally essential elements in normal and diabetic individuals. Diabetes.

[79] Qiu Q., Zhang F., Zhu W., Wu J., Liang M. (2017). Copper in Diabetes Mellitus: A Meta-Analysis and Systematic Review of Plasma and Serum Studies. Biol. Trace Elem. Res..

[80] Tanaka A., Kaneto H., Miyatsuka T., Yamamoto K., Yoshiuchi K., Yamasaki Y., Shimomura I., Matsuoka T.A., Matsuhisa M. (2009). Role of copper ion in the pathogenesis of type 2 diabetes. Endocr. J..

[81] Hamann I., Petroll K., Grimm L., Hartwig A., Klotz L.O. (2014). Insulin-like modulation of Akt/FoxO signaling by copper ions is independent of insulin receptor. Arch. Biochem. Biophys..

[82] Ostrakhovitch E.A., Lordnejad M.R., Schliess F., Sies H., Klotz L.O. (2002). Copper ions strongly activate the phosphoinositide-3-kinase/Akt pathway independent of the generation of reactive oxygen species. Arch. Biochem. Biophys..

[83] Schmoll D., Walker K.S., Alessi D.R., Grempler R., Burchell A., Guo S., Walther R., Unterman T.G. (2000). Regulation of glucose-6-phosphatase gene expression by protein kinase Bα and the forkhead transcription factor FKHR. Evidence for insulin response unit-dependent and -independent effects of insulin on promoter activity. J. Biol. Chem..

[84] Kim J.H., Cho H., Ryu S.E., Choi M.U. (2000). Effects of metal ions on the activity of protein tyrosine phosphatase VHR: Highly potent and reversible oxidative inactivation by Cu^2+^ ion. Arch. Biochem. Biophys..

[85] Goldstein B.J., Ahmad F., Ding W., Li P.M., Zhang W.R. (1998). Regulation of the insulin signalling pathway by cellular protein-tyrosine phosphatases. Mol. Cell. Biochem..

[86] Wei X.B., Guo L., Liu Y., Zhou S.R., Liu Y., Dou X., Du S.Y., Ding M., Peng W.Q., Qian S.W. (2017). Synthesis of cytochrome c oxidase 1 (SCO1) inhibits insulin sensitivity by decreasing copper levels in adipocytes. Biochem. Biophys. Res. Commun..

[87] Tolbert M.E., Kamalu J.A., Draper G.D. (1981). Effects of cadmium, zinc, copper and manganese on hepatic parenchymal cell gluconeogenesis. J. Environ. Sci. Health B.

[88] Grasso G., Salomone F., Tundo G.R., Pappalardo G., Ciaccio C., Spoto G., Pietropaolo A., Coletta M., Rizzarelli E. (2012). Metal ions affect insulin-degrading enzyme activity. J. Inorg. Biochem..

[89] Bellia F., Lanza V., Ahmed I.M.M., Garcia-Vinuales S., Veiss E., Arizzi M., Calcagno D., Milardi D., Grasso G. (2019). Site directed mutagenesis of insulin-degrading enzyme allows singling out the molecular basis of peptidase versus E1-like activity: The role of metal ions. Metallomics.

[90] Grasso G., Pietropaolo A., Spoto G., Pappalardo G., Tundo G.R., Ciaccio C., Coletta M., Rizzarelli E. (2011). Copper(I) and copper(II) inhibit Aβ peptides proteolysis by insulin-degrading enzyme differently: Implications for metallostasis alteration in Alzheimer’s disease. Chemistry.

[91] Maianti J.P., McFedries A., Foda Z.H., Kleiner R.E., Du X.Q., Leissring M.A., Tang W.J., Charron M.J., Seeliger M.A., Saghatelian A. (2014). Anti-diabetic activity of insulin-degrading enzyme inhibitors mediated by multiple hormones. Nature.

[92] Yuk I.H., Zhang J.D., Ebeling M., Berrera M., Gomez N., Werz S., Meiringer C., Shao Z., Swanberg J.C., Lee K.H. (2014). Effects of copper on CHO cells: Insights from gene expression analyses. Biotechnol. Prog..

[93] Wu Z., Zhang W., Kang Y.J. (2019). Copper affects the binding of HIF-1α to the critical motifs of its target genes. Metallomics.

[94] Ishida S., Andreux P., Poitry-Yamate C., Auwerx J., Hanahan D. (2013). Bioavailable copper modulates oxidative phosphorylation and growth of tumors. Proc. Natl. Acad. Sci. USA.

[95] Ramchandani D., Berisa M., Tavarez D.A., Li Z., Miele M., Bai Y., Lee S.B., Ban Y., Dephoure N., Hendrickson R.C. (2021). Copper depletion modulates mitochondrial oxidative phosphorylation to impair triple negative breast cancer metastasis. Nat. Commun..

[96] Jensen E.L., Gonzalez-Ibanez A.M., Mendoza P., Ruiz L.M., Riedel C.A., Simon F., Schuringa J.J., Elorza A.A. (2019). Copper deficiency-induced anemia is caused by a mitochondrial metabolic reprograming in erythropoietic cells. Metallomics.

[97] Ruiz L.M., Jensen E.L., Rossel Y., Puas G.I., Gonzalez-Ibanez A.M., Bustos R.I., Ferrick D.A., Elorza A.A. (2016). Non-cytotoxic copper overload boosts mitochondrial energy metabolism to modulate cell proliferation and differentiation in the human erythroleukemic cell line K562. Mitochondrion.

[98] Rozenberg J.M., Kamynina M., Sorokin M., Zolotovskaia M., Koroleva E., Kremenchutckaya K., Gudkov A., Buzdin A., Borisov N. (2022). The Role of the Metabolism of Zinc and Manganese Ions in Human Cancerogenesis. Biomedicines.

[99] Hurley L.S., Keen C.L., Baly D.L. (1984). Manganese deficiency and toxicity: Effects on carbohydrate metabolism in the rat. Neurotoxicology.

[100] Flores C.R., Puga M.P., Wrobel K., Garay Sevilla M.E., Wrobel K. (2011). Trace elements status in diabetes mellitus type 2: Possible role of the interaction between molybdenum and copper in the progress of typical complications. Diabetes Res. Clin. Pract..

[101] Bresciani G., Cruz I.B., de Paz J.A., Cuevas M.J., González-Gallego J. (2013). The MnSOD Ala16Val SNP: Relevance to human diseases and interaction with environmental factors. Free Radic. Res..

[102] Lee S.H., Jouihan H.A., Cooksey R.C., Jones D., Kim H.J., Winge D.R., McClain D.A. (2013). Manganese supplementation protects against diet-induced diabetes in wild type mice by enhancing insulin secretion. Endocrinology.

[103] Burlet E., Jain S.K. (2013). Manganese supplementation reduces high glucose-induced monocyte adhesion to endothelial cells and endothelial dysfunction in Zucker diabetic fatty rats. J. Biol. Chem..

[104] Baly D.L., Curry D.L., Keen C.L., Hurley L.S. (1984). Effect of manganese deficiency on insulin secretion and carbohydrate homeostasis in rats. J. Nutr..

[105] Baly D.L., Curry D.L., Keen C.L., Hurley L.S. (1985). Dynamics of insulin and glucagon release in rats: Influence of dietary manganese. Endocrinology.

[106] Bryan M.R., Uhouse M.A., Nordham K.D., Joshi P., Rose D.I.R., O’Brien M.T., Aschner M., Bowman A.B. (2018). Phosphatidylinositol 3 kinase (PI3K) modulates manganese homeostasis and manganese-induced cell signaling in a murine striatal cell line. Neurotoxicology.

[107] Bryan M.R., Nordham K.D., Rose D.I.R., O’Brien M.T., Joshi P., Foshage A.M., Gonçalves F.M., Nitin R., Uhouse M.A., Aschner M. (2020). Manganese Acts upon Insulin/IGF Receptors to Phosphorylate AKT and Increase Glucose Uptake in Huntington’s Disease Cells. Mol. Neurobiol..

[108] Ueda M., Robinson F.W., Smith M.M., Kono T. (1984). Effects of divalent cations on the regulation of insulin-sensitive glucose transport and cAMP phosphodiesterase in adipocytes. Insulin-like effects of divalent cations. J. Biol. Chem..

[109] Baly D.L., Schneiderman J.S., Garcia-Welsh A.L. (1990). Effect of manganese deficiency on insulin binding, glucose transport and metabolism in rat adipocytes. J. Nutr..

[110] Nicastro R., Gaillard H., Zarzuela L., Péli-Gulli M.P., Fernández-García E., Tomé M., García-Rodríguez N., Durán R.V., De Virgilio C., Wellinger R.E. (2022). Manganese is a physiologically relevant TORC1 activator in yeast and mammals. eLife.

[111] Mao Z., Zhang W. (2018). Role of mTOR in Glucose and Lipid Metabolism. Int. J. Mol. Sci..

[112] Malthankar G.V., White B.K., Bhushan A., Daniels C.K., Rodnick K.J., Lai J.C.K. (2004). Differential lowering by manganese treatment of activities of glycolytic and tricarboxylic acid (TCA) cycle enzymes investigated in neuroblastoma and astrocytoma cells is associated with manganese-induced cell death. Neurochem. Res..

[113] Wimhurst J.M., Manchester K.L. (1973). Effects of manganese on the activity of glycolytic and gluconeogenic enzymes in the perfused rat liver. FEBS Lett..

[114] Delmastro-Greenwood M.M., Votyakova T., Goetzman E., Marre M.L., Previte D.M., Tovmasyan A., Batinic-Haberle I., Trucco M.M., Piganelli J.D. (2013). Mn porphyrin regulation of aerobic glycolysis: Implications on the activation of diabetogenic immune cells. Antioxid. Redox Signal..

[115] Zwingmann C., Leibfritz D., Hazell A.S. (2003). Energy metabolism in astrocytes and neurons treated with manganese: Relation among cell-specific energy failure, glucose metabolism, and intercellular trafficking using multinuclear NMR-spectroscopic analysis. J. Cereb. Blood Flow Metab..

[116] Zhang F., Xu Z., Gao J., Xu B., Deng Y. (2008). In vitro effect of manganese chloride exposure on energy metabolism and oxidative damage of mitochondria isolated from rat brain. Environ. Toxicol. Pharmacol..

[117] Kaur G., Kumar V., Arora A., Tomar A., Ashish, Sur R., Dutta D. (2017). Affected energy metabolism under manganese stress governs cellular toxicity. Sci. Rep..

[118] Mittler R., Vanderauwera S., Suzuki N., Miller G., Tognetti V.B., Vandepoele K., Gollery M., Shulaev V., Van Breusegem F. (2011). ROS Signaling: The New Wave?. Trends Plant Sci..

[119] Lee S., Tak E., Lee J., Rashid M.A., Murphy M.P., Ha J., Kim S.S. (2011). Mitochondrial H_2_O_2_ generated from electron transport chain complex I stimulates muscle differentiation. Cell Res..

[120] Raffield L.M., Louie T., Sofer T., Jain D., Ipp E., Taylor K.D., Papanicolaou G.J., Avilés-Santa L., Lange L.A., Laurie C.C. (2017). Genome-wide association study of iron traits and relation to diabetes in the Hispanic Community Health Study/Study of Latinos (HCHS/SOL): Potential genomic intersection of iron and glucose regulation?. Hum. Mol. Genet..

[121] Simcox J.A., McClain D.A. (2013). Iron and diabetes risk. Cell Metab..

[122] Fernández-Real J.M., Manco M. (2014). Effects of iron overload on chronic metabolic diseases. Lancet Diabetes Endocrinol..

[123] Liu Q., Sun L., Tan Y., Wang G., Lin X., Cai L. (2009). Role of iron deficiency and overload in the pathogenesis of diabetes and diabetic complications. Curr. Med. Chem..

[124] Kranz R.G., Richard-Fogal C., Taylor J.S., Frawley E.R. (2009). Cytochrome c biogenesis: Mechanisms for covalent modifications and trafficking of heme and for heme-iron redox control. Microbiol. Mol. Biol. Rev..

[125] Hagler L., Askew E.W., Neville J.R., Mellick P.W., Coppes R.I., Lowder J.F. (1981). Influence of dietary iron deficiency on hemoglobin, myoglobin, their respective reductases, and skeletal muscle mitochondrial respiration. Am. J. Clin. Nutr..

[126] Frise M.C., Holdsworth D.A., Johnson A.W., Chung Y.J., Curtis M.K., Cox P.J., Clarke K., Tyler D.J., Roberts D.J., Ratcliffe P.J. (2022). Abnormal whole-body energy metabolism in iron-deficient humans despite preserved skeletal muscle oxidative phosphorylation. Sci. Rep..

[127] Chung Y.J., Swietach P., Curtis M.K., Ball V., Robbins P.A., Lakhal-Littleton S. (2020). Iron-Deficiency Anemia Results in Transcriptional and Metabolic Remodeling in the Heart Toward a Glycolytic Phenotype. Front. Cardiovasc. Med..

[128] Stugiewicz M., Tkaczyszyn M., Kasztura M., Banasiak W., Ponikowski P., Jankowska E.A. (2016). The influence of iron deficiency on the functioning of skeletal muscles: Experimental evidence and clinical implications. Eur. J. Heart Fail..

[129] Quail E.A., Yeoh G.C. (1995). The effect of iron status on glyceraldehyde 3-phosphate dehydrogenase expression in rat liver. FEBS Lett..

[130] Li J., Jia L., Ma W., Feng Y., Yu H., Du H. (2022). Dietary iron modulates hepatic glucose homeostasis via regulating gluconeogenesis. J. Nutr. Biochem..

[131] Ohira Y., Chen C.S., Hegenauer J., Saltman P. (1983). Adaptations of lactate metabolism in iron-deficient rats. Proc. Soc. Exp. Biol. Med..

[132] Henderson S.A., Dallman P.R., Brooks G.A. (1986). Glucose turnover and oxidation are increased in the iron-deficient anemic rat. Am. J. Physiol..

[133] Liu J., Li Q., Yang Y., Ma L. (2020). Iron metabolism and type 2 diabetes mellitus: A meta-analysis and systematic review. J. Diabetes Investig..

[134] He J., Fang A., Yu S., Shen X., Li K. (2020). Dietary Nonheme, Heme, and Total Iron Intake and the Risk of Diabetes in Adults: Results from the China Health and Nutrition Survey. Diabetes Care.

[135] Yang J.H., Downes K., Howson J.M., Nutland S., Stevens H.E., Walker N.M., Todd J.A. (2011). Evidence of association with type 1 diabetes in the SLC11A1 gene region. BMC Med. Genet..

[136] Kavian Z., Sargazi S., Majidpour M., Sarhadi M., Saravani R., Shahraki M., Mirinejad S., Heidari Nia M., Piri M. (2023). Association of SLC11A1 polymorphisms with anthropometric and biochemical parameters describing Type 2 Diabetes Mellitus. Sci. Rep..

[137] Tilbrook L. (2004). Cross talk between iron metabolism and diabetes. Ann. Clin. Biochem..

[138] Dandona P., Hussain M.A., Varghese Z., Politis D., Flynn D.M., Hoffbrand A.V. (1983). Insulin resistance and iron overload. Ann. Clin. Biochem..

[139] Hua N.W., Stoohs R.A., Facchini F.S. (2001). Low iron status and enhanced insulin sensitivity in lacto-ovo vegetarians. Br. J. Nutr..

[140] Messner D.J., Rhieu B.H., Kowdley K.V. (2013). Iron overload causes oxidative stress and impaired insulin signaling in AML-12 hepatocytes. Dig. Dis. Sci..

[141] Fargion S., Dongiovanni P., Guzzo A., Colombo S., Valenti L., Fracanzani A.L. (2005). Iron and insulin resistance. Aliment. Pharmacol. Ther..

[142] Dongiovanni P., Valenti L., Ludovica Fracanzani A., Gatti S., Cairo G., Fargion S. (2008). Iron depletion by deferoxamine up-regulates glucose uptake and insulin signaling in hepatoma cells and in rat liver. Am. J. Pathol..

[143] Mehdad A., Campos N.A., Arruda S.F., Siqueira E.M. (2015). Iron Deprivation May Enhance Insulin Receptor and Glut4 Transcription in Skeletal Muscle of Adult Rats. J. Nutr. Health Aging.

[144] Zhao X., Ma Y., Shi M., Huang M., Xin J., Ci S., Chen M., Jiang T., Hu Z., He L. (2023). Excessive iron inhibits insulin secretion via perturbing transcriptional regulation of SYT7 by OGG1. Cell. Mol. Life Sci..

[145] Blesia V., Patel V.B., Al-Obaidi H., Renshaw D., Zariwala M.G. (2021). Excessive Iron Induces Oxidative Stress Promoting Cellular Perturbations and Insulin Secretory Dysfunction in MIN6 Beta Cells. Cells.

[146] Li W., Feng Q., Wang C., Yin Z., Li X., Li L. (2022). LncXIST Facilitates Iron Overload and Iron Overload-Induced Islet Beta Cell Injury in Type 2 Diabetes through miR-130a-3p/ALK2 Axis. Comput. Intell. Neurosci..

[147] Deng L., Mo M.Q., Zhong J., Li Z., Li G., Liang Y. (2023). Iron overload induces islet β cell ferroptosis by activating ASK1/P-P38/CHOP signaling pathway. PeerJ.

[148] Hansen J.B., Tonnesen M.F., Madsen A.N., Hagedorn P.H., Friberg J., Grunnet L.G., Heller R.S., Nielsen A., Størling J., Baeyens L. (2012). Divalent metal transporter 1 regulates iron-mediated ROS and pancreatic β cell fate in response to cytokines. Cell Metab..

[149] Santos M., Anderson C.P., Neschen S., Zumbrennen-Bullough K.B., Romney S.J., Kahle-Stephan M., Rathkolb B., Gailus-Durner V., Fuchs H., Wolf E. (2020). Irp2 regulates insulin production through iron-mediated Cdkal1-catalyzed tRNA modification. Nat. Commun..

[150] Ashok A., Singh N. (2018). Prion protein modulates glucose homeostasis by altering intracellular iron. Sci. Rep..

[151] Higashida K., Takeuchi N., Inoue S., Hashimoto T., Nakai N. (2020). Iron deficiency attenuates catecholamine-stimulated lipolysis via downregulation of lipolysis-related proteins and glucose utilization in 3T3-L1 adipocytes. Mol. Med. Rep..

[152] Fillebeen C., Lam N.H., Chow S., Botta A., Sweeney G., Pantopoulos K. (2020). Regulatory Connections between Iron and Glucose Metabolism. Int. J. Mol. Sci..

[153] De Meyts P., Feingold K.R., Anawalt B., Blackman M.R., Boyce A., Chrousos G., Corpas E., de Herder W.W., Dhatariya K., Dungan K. (2000). The Insulin Receptor and Its Signal Transduction Network. Endotext.

[154] Chang L., Chiang S.H., Saltiel A.R. (2004). Insulin signaling and the regulation of glucose transport. Mol. Med..

[155] De Romaña D.L., Olivares M., Uauy R., Araya M. (2011). Risks and benefits of copper in light of new insights of copper homeostasis. J. Trace Elem. Med. Biol..

[156] Dallinger R. (1994). Invertebrate organisms as biological indicators of heavy metal pollution. Appl. Biochem. Biotechnol..

[157] Michalke B., Fernsebner K. (2014). New insights into manganese toxicity and speciation. J. Trace Elem. Med. Biol..

[158] Lu J., Gong D., Choong S., Xu H., Chan Y., Chen X., Fitzpatrick S., Glyn-Jones S., Zhang S., Nakamura T. (2010). Copper (II)-selective chelation improves function and antioxidant defences in cardiovascular tissues of rats as a model of diabetes: Comparisons between triethylenetetramine and three less copper-selective transition-metal-targeted treatments. Diabetologia.

[159] Gong D., Lu J., Chen X., Reddy S., Crossman D., Glyn-Jones S., Choong Y.-S., Kennedy J., Barry B., Zhang S. (2008). A copper (II)-selective chelator ameliorates diabetes-evoked renal fibrosis and albuminuria, and suppresses pathogenic TGF-β activation in the kidneys of rats used as a model of diabetes. Diabetologia.

[160] Song E., Vu V., Varin T.V., Botta A., Marette A., Sweeney G. (2022). Copper fabric improves the metabolic profile of obese mice: Potential role of the gut microbiota. Basic Clin. Pharmacol. Toxicol..

[161] Sun Q., Van Dam R.M., Willett W.C., Hu F.B. (2009). Prospective study of zinc intake and risk of type 2 diabetes in women. Diabetes Care.

[162] Laouali N., MacDonald C.-J., Shah S., El Fatouhi D., Mancini F.R., Fagherazzi G., Boutron-Ruault M.-C. (2021). Dietary copper/zinc ratio and type 2 diabetes risk in women: The E3N cohort study. Nutrients.

[163] Gunasekara P., Hettiarachchi M., Liyanage C., Lekamwasam S. (2011). Effects of zinc and multimineral vitamin supplementation on glycemic and lipid control in adult diabetes. Diabetes Metab. Syndr. Obes. Targets Ther..

[164] Zhu K., Nie S., Li C., Huang J., Hu X., Li W., Gong D., Xie M. (2013). Antidiabetic and pancreas-protective effects of zinc threoninate chelate in diabetic rats may be associated with its antioxidative stress ability. Biol. Trace Elem. Res..

[165] Karatug A., Kaptan E., Bolkent S., Mutlu O., Yanardag R. (2013). Alterations in kidney tissue following zinc supplementation to STZ-induced diabetic rats. J. Trace Elem. Med. Biol..

[166] Cutler P. (1989). Deferoxamine therapy in high-ferritin diabetes. Diabetes.

[167] Cooksey R.C., Jones D., Gabrielsen S., Huang J., Simcox J.A., Luo B., Soesanto Y., Rienhoff H., Dale Abel E., McClain D.A. (2010). Dietary iron restriction or iron chelation protects from diabetes and loss of β-cell function in the obese (ob/ob lep−/−) mouse. Am. J. Physiol. Endocrinol. Metab..

[168] Tajima S., Ikeda Y., Sawada K., Yamano N., Horinouchi Y., Kihira Y., Ishizawa K., Izawa-Ishizawa Y., Kawazoe K., Tomita S. (2012). Iron reduction by deferoxamine leads to amelioration of adiposity via the regulation of oxidative stress and inflammation in obese and type 2 diabetes KKAy mice. Am. J. Physiol. Endocrinol. Metab..

[169] Jirakittidul P., Sirichotiyakul S., Ruengorn C., Siripenpong S., Imruetaicharoenchok A., Wiriyasirivaj B. (2019). Iron supplementation in non-anemic pregnancy and risk of developing gestational diabetes mellitus. J. Endocrinol. Metab..

[170] Burlet E., Jain S.K. (2017). Manganese supplementation increases adiponectin and lowers ICAM-1 and creatinine blood levels in Zucker type 2 diabetic rats, and downregulates ICAM-1 by upregulating adiponectin multimerization protein (DsbA-L) in endothelial cells. Mol. Cell. Biochem..

[171] Du S., Wu X., Han T., Duan W., Liu L., Qi J., Niu Y., Na L., Sun C. (2018). Dietary manganese and type 2 diabetes mellitus: Two prospective cohort studies in China. Diabetologia.

