# Supplementary material for: The Link between Trace Metal Elements and Glucose Metabolism: Evidence from Zinc, Copper, Iron, and Manganese-Mediated Metabolic Regulation
Source: Metabolites. 2023 Oct 2;13(10):1048. doi: 10.3390/metabo13101048 (PMC10608713; doi:10.3390/metabo13101048)
Supplement: Supplementary file 1 [file metabolites-13-01048-s001.zip › metabolites-2588821-supplementary.pdf]

metabolites

MDPI

Supplementary Table S1. Implications of tr01ace metal elements in diabetic therapy.

Metal	Method	Samples	Results	References
Copper	Oral tetratiomolybdate intake (TETA, copper chelating agent)	C57BL/KsJ-db/db mice	TETA improves insulin resistance and restored glucose intolerance, reduces serum triglyceride levels.	[80]
	Oral TETA intake	Wistar rats, injection of STZ,	TETA reduces diabetic complications damage, increases the activity of SOD.	[158]
	Oral TETA intake	Wistar rats, injection of STZ,	TETA attenuates diabetic kidney disease by suppressing the activity of activation of TGF-β signaling.	[159]
	Pretreat with $CuCl_2·2H_2O$	3T3-L1 cells	High copper level can restore the activity of insulin signaling by down-regulating PTEN protein in adipocytes.	[86]
	Copper-containing fabric bedding	High fat, high cholesterol diet induced obesity mice	With contrary of control, mouse housed on copper-containing fabric bedding has less Reduces body weight, increases glucose tolerance.	[160]
	Foods with high level zinc	U.S. T2D Women	In part, higher zinc intake can diminish the risk of T2D.	[161]
	Foods contain zinc	French Women,<br>a prospective cohort study	Obesity women with zinc intake >8mg/day and low Cu/Zn ratio reduces the risk of T2D.	[162]

**Zinc**	Oral zinc sulfate intake	T2D patients,<br><br>adult-onset	Zinc supplementation improves glucose control, changes serum cholesterol level and the ratio of cholesterol/high-density lipoprotein. Zinc sulfate and multivitamin/mineral has the potential to protect diabetes.	[163]
	Oral intake of zinc threoninate chelate	Wistar rats,<br><br>STZ injection	Zinc threoninate chelate reduces serum glucose concentration, increases insulin levels and the activation of Cu/Zn-SOD.	[164]
	Oral zinc sulfate intake	Swiss albino rats,<br><br>STZ injection	Zinc sulfate increases GSH levels in kidney, enhances antioxidant potential, defenses kidney damage.	[165]
**Iron**	Oral DFO intake	White diabetic patients with high levels of ferritin	To high-ferritin diabetic patients, DFO has a positive effect to reduce glucose, triglyceride and HbA1c.	[166]
	Dietary iron-restriction (35 mg/kg iron);	Ob/Ob mice with T2D	Iron-restricted diet can ameliorate insulin sensitivity and β-islet cell function.	[167]
	Oral DFO intake	KKAy mice	Iron chelator decreases macrophage infiltration into fat, leading to lower inflammatory cytokines and oxidative stress, which ameliorates a vicious cycle in obesity.	[168]
	Oral ferrous fumarate intake	Non-anemic pregnant women	Early iron supplementation increases the risk of gestational diabetes.	[169]
**Manganese**	Injection of 12 mg/kg $MnCl_2$	C57BL/6J mice with a high-fat diet	Mn treatment increases insulin secretory capacity of β-islet cell and the activity of Mn-SOD.	[102]
	Gavage $MnCl_2$ solution or cells pretreated with $MnCl_2$	Zucker diabetic fatty rats	Mn supplementation increases adiponectin and protect endothelial dysfunction in diabetes.	[103]

	Gavage $MnCl_2$ solution or cells pretreated with $MnCl_2$	Zucker diabetic fatty rats	Mn supplements can decrease ICAM-1 expression and ROS, reduces adhesion of monocytes to endothelial cells, diminishes the risk of vascular disease in diabetic animals	[170]
	Consume food with Mn	Chinese adults with T2D	Mn intake and the incidence of diabetes have a negative association.	[171]

T2D: Diabetes mellitus type 2; TETA, triethylenetetramine; STZ, streptozotocin; SOD, superoxide dismutase; GSH, glutathione; DFO, deferoxamine.
